# Now on display: a gallery of group II intron structures at different stages of catalysis

**DOI:** 10.1186/1759-8753-4-14

**Published:** 2013-05-01

**Authors:** Marco Marcia, Srinivas Somarowthu, Anna Marie Pyle

**Affiliations:** 1 Department of Molecular, Cellular and Developmental Biology, Yale University, New Haven, CT 06511, USA; 2 Department of Chemistry, Yale University, New Haven, CT 06511, USA; 3 Howard Hughes Medical Institute, Chevy Chase, MD 20815, USA

**Keywords:** Retrotransposition, Spliceosome, X-ray structures, RNA catalysis, Metal ions

## Abstract

Group II introns are mobile genetic elements that self-splice and retrotranspose into DNA and RNA. They are considered evolutionary ancestors of the spliceosome, the ribonucleoprotein complex essential for pre-mRNA processing in higher eukaryotes. Over a 20-year period, group II introns have been characterized first genetically, then biochemically, and finally by means of X-ray crystallography. To date, 17 crystal structures of a group II intron are available, representing five different stages of the splicing cycle. This review provides a framework for classifying and understanding these new structures in the context of the splicing cycle. Structural and functional implications for the spliceosome are also discussed.

## Review

Group II introns are mobile ribozymes capable of self-splicing and retrotransposition [1]. As retrotransposable elements, group II introns have invaded the genomes of most life forms and enhanced genomic diversity in all domains of life. In this way, they have played a crucial role in the evolution of modern organisms [2,3]. At the present time, they remain important in archaea, bacteria, and unicellular and multicellular eukaryotes because they ensure the correct expression of certain housekeeping genes and because they hinder the distribution of other harmful mobile genetic elements [4,5]. Of particular interest to the field of RNA processing, group II introns are considered evolutionary ancestors of the spliceosome, which is the ribonucleoprotein complex essential for pre-mRNA processing in higher eukaryotes, including humans [6-8]. Finally, group II introns are potentially useful medical tools, because they can be artificially reprogrammed to insert into desired DNA or RNA sites [9-11]. Consequently, they are macromolecules of great microbiological, biotechnological and pharmacological interest.

**Figure 1:**
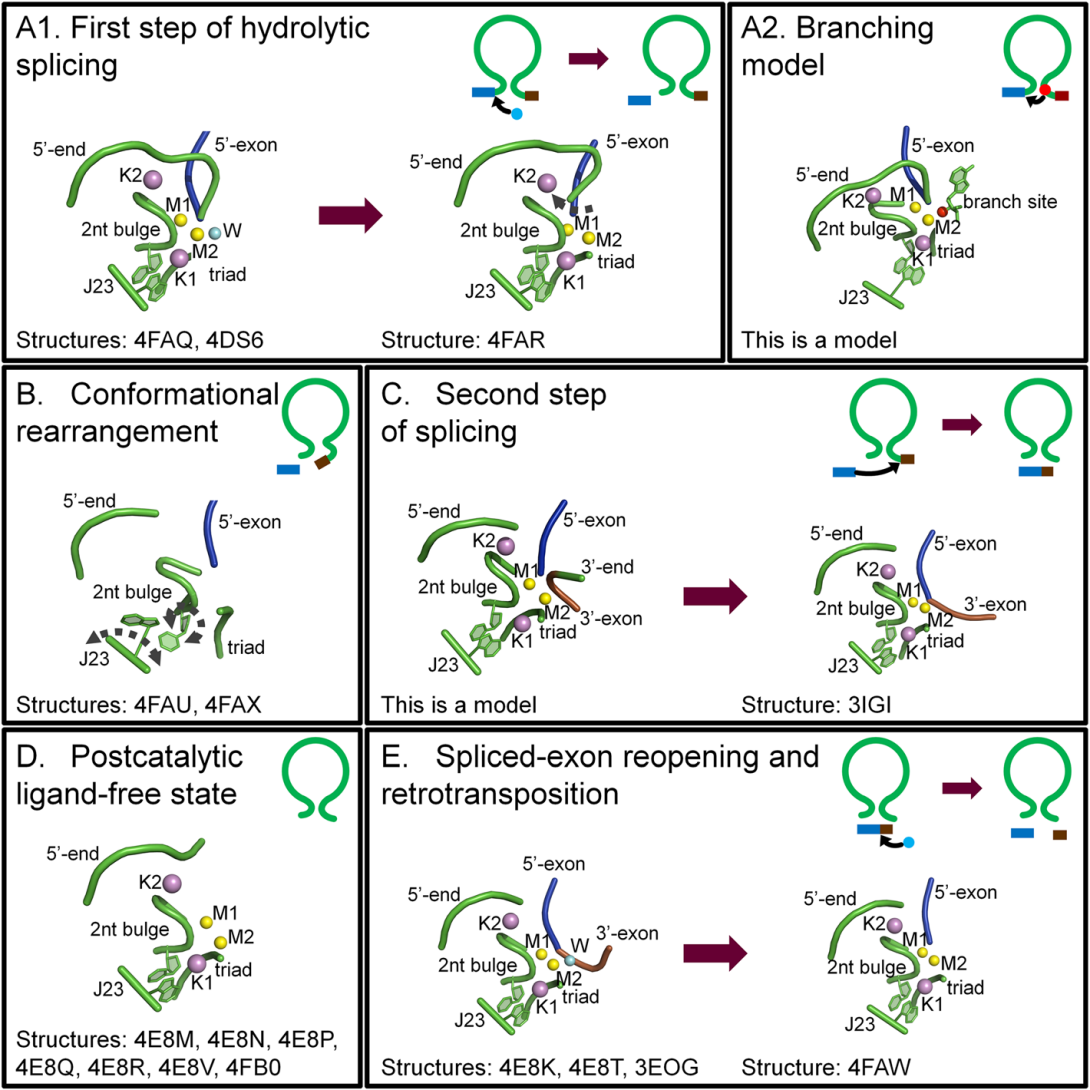
**Group II intron splicing cycle.** Upon transcription, group II introns fold into an active structure, which catalyzes the first splicing step by hydrolysis (**A1**) or transesterification (branching, **A2**). Successively, the intron rearranges its active site conformation (**B**) to recruit the 3′-splice junction into the active site. The 3′-splice junction is then subject to nucleophilic attack by the free 5′-exon (second splicing step, **C**) to form ligated exons and a ligand-free intron (**D**). The latter is still a highly active ribozyme. *In vitro* it tends to rehydrolyze ligated exons by SER, following a reaction mechanism that shares the same stereochemistry as the first step of reverse splicing, by which the intron retrotransposes into DNA or RNA targets (**E**). In the top right corner of each panel is a schematic of the reaction (5′-exon in blue, intron in green, 3′-exon in brown, nucleophilic water molecules in cyan spheres, branch site nucleophile in red spheres, nucleophilic attacks as black arrows). In the middle of each panel are snapshots of the active sites from representative structures or models (same color code, ions M1-M2 as yellow and K1-K2 as violet spheres, conformational changes as grey dotted arrows). At the bottom of each panel are the Protein Data Bank identifiers of all structures corresponding to each stage of the cycle, or the phrase ‘This is a model’ for those states that were not obtained crystallographically but by modeling. SER: spliced-exon reopening.

Group II introns catalyze splicing in a series of S_N_2 reactions (Figure 1). Briefly, in the first splicing step, a water molecule or the 2′-OH group of a bulged adenosine in D6 attacks the 5′-splice junction, forming an intron/3′-exon intermediate. After the first splicing step, the intron is believed to rearrange and prepare for the second splicing step [12]. During this final step, the 5′-exon performs a nucleophilic addition to the 3′-splice junction, releasing ligated exons and the excised intron in a linear or lariat form. Finally, the lifecycle of a group II intron can also include reverse splicing of the excised intron into target positions within genomic DNA of the host organism, along with retrotranscription via an intron-encoded maturase, culminating in a process known as retrohoming or retrotransposition. At a molecular level, the reverse splicing reaction involves the same target recognition elements and proceeds with the same stereochemistry as the so-called spliced-exon reopening (SER) reaction, by which the free intron specifically recognizes and cleaves the ligated exons *in vitro*[13-15]. Therefore, SER is considered a biochemical mimic of retrotransposition.

**Figure 2:**
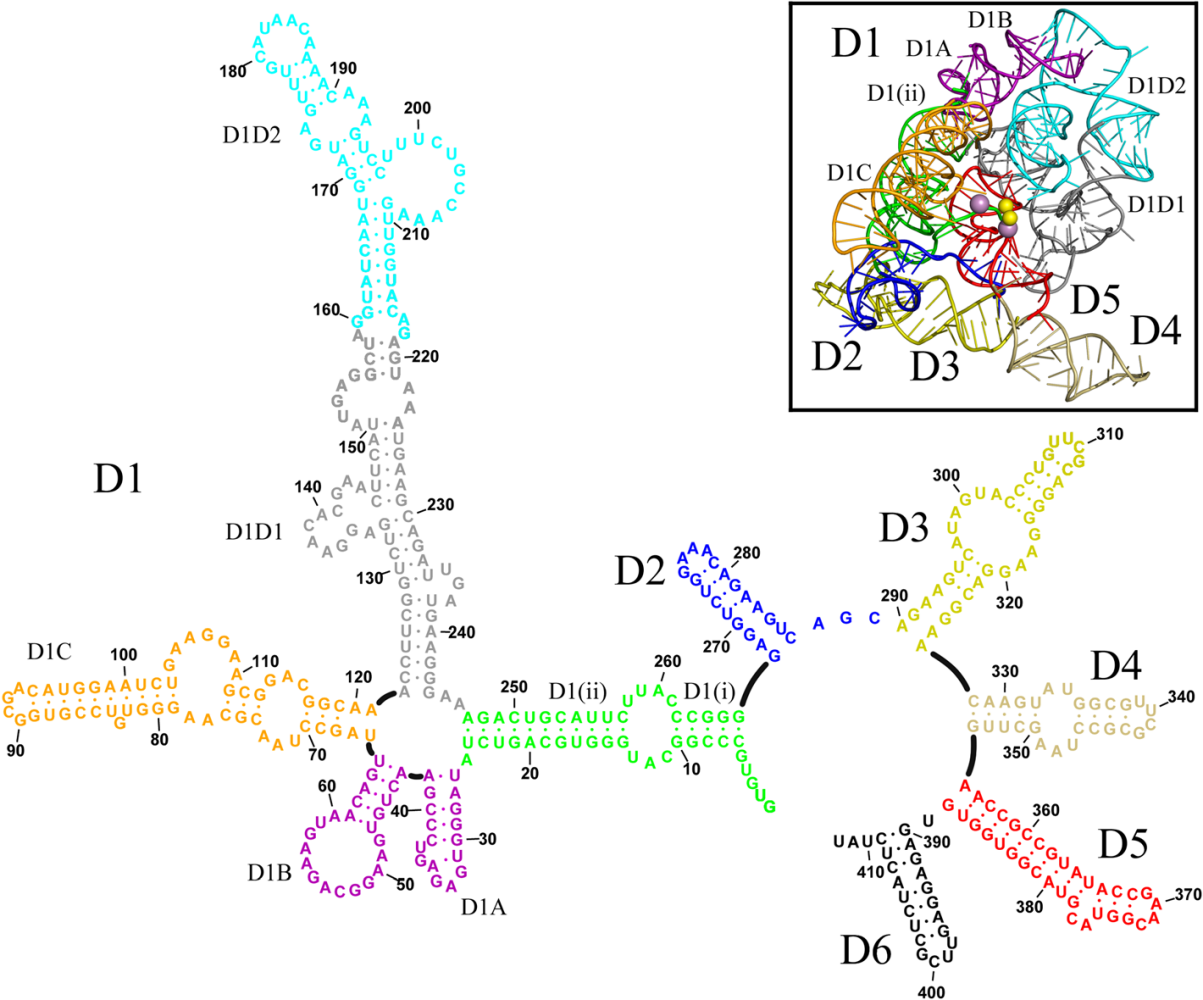
**Secondary and tertiary structure of group II introns.** The diagram shows the secondary structure of the *Oceanobacillus iheyensis* group II intron. The structure is characterized by six domains (D1 to D6) and D1 is formed by smaller subdomains (D1(i), D1(ii), D1A, D1B, D1C, D1D1, and D1D2). Each subdomain of D1 and D2, D3, D4, D5 and D6 are depicted in different colors. The inset shows the tertiary structure of the *Oceanobacillus iheyensis* group II intron (same color code). The four active site metal ions are represented as yellow (Mg^2+^) and violet (K^+^) spheres, respectively. The figure was adapted from [26].

The functionality of group II introns is mediated primarily by their intricate and stable three-dimensional structure. Historically, the structure of group II introns was elucidated throughout a 20-year long, stepwise process. Initially, phylogenetic studies showed that, despite their relatively poor sequence conservation, all group II introns share a common secondary structure and are composed of six domains (D1 through D6, Figure 2) [16-20]. Three major classes of group II introns have been identified and designated IIA, IIB and IIC. The group IIA and IIB classes are approximately 900 nt long and are found in bacteria, archaea, mitochondria and chloroplasts, while the introns belonging to the group IIC class are shorter (approximately 400 nt) and they are present exclusively in prokaryotes, representing the most primitive lineage of group II intron ribozymes [21]. More recent work has indicated that additional families of group II introns exist, and as new sequences are discovered, useful new classifications are being developed [16]. Over time, a series of biochemical experiments performed primarily on the group IIB ai5γ intron from yeast mitochondria (reviewed in [4]), on the group IIA and IIB introns from the brown alga *Pylaiella littoralis*[22], and on the group IIA intron Ll.LtrB from *Lactococcus lactis*[23] led to the definition of tertiary contacts and to the design of tertiary structure maps [23-25], which provided a concrete understanding of functional intron architecture. Ultimately, a breakthrough in understanding group II intron structure-function relationships was made possible by a crystal structure of the self-spliced form of the group IIC intron from *Oceanobacillus iheyensis* (*O.i.*) [26]. The crystal structure demonstrated how D1 of the intron forms a compact scaffold, which encloses the other intron domains, and presents the exon recognition elements (exon binding sites, EBSs). By contrast, D2 and D4 project away from the intron core, enabling them to encode sequence insertions and open reading frames. D3 acts as an interaction hub [27] further stabilizing the structure thanks to its characteristic internal loop and conserved S-turn. Most importantly, the highly conserved D5 forms the active site, where the catalytic triad (C358-G359-C360, the numbering is for the *O.i.* group II intron), the two-nucleotide bulge (A376-C377), and the J2/3 junction (A287-G288-C289) join into a major-groove triple helix. Only D6, which contains the branch-site adenosine (A406) and which connects to the 3′-splice site, could not be visualized crystallographically because of its intrinsic flexibility [21,28].

A detailed description of the structural features specific to each domain, and of tertiary interactions amongst the domains, has already been reported [21]. However, a wealth of new structural information about group II introns has recently become available through a series of new crystallographic studies [29-31]. For the first time, these structures depict the intron at different stages of the splicing cycle (Figure 1, Table 1), revealing the positions and the roles of critical functional elements, including reactants and substrates before and after catalysis, and in multiple alternative conformations. Moreover, some of the new crystal structures also define the position and identity of key metal ions, demonstrating how different types of metals stabilize the intron structure and participate in catalysis [30].

**Table 1:** Available 3-D X-ray structures of the group II intron

**PDB id**	**Resolution (Å)**	**Metals**	**Splicing stage**	**Activity**	**Construct**	**Reference**
4DS6	3.64	NH_4_^+^/Mg^2+^	5′-exon hydrolysis (pre)	No (active site mutant)	OiD1-6-G359A	[29]
4FAQ	3.11	K^+^/Ca^2+^	5′-exon hydrolysis (pre)	No (nonfunctional divalent ion)	Oi5eD1-5	[30]
4FAR	2.86	K^+^/Mg^2+^	5′-exon hydrolysis (post)	Yes	Oi5eD1-5	[30]
4FAU	2.87	Li^+^/Mg^2+^	Intermediate	Partial (nonfunctional monovalent ion)	Oi5eD1-5	[30]
3IGI^a^	3.13	K^+^/Mg^2+^	Postcatalytic ligand-bound	Yes	OiD1-6	[26,27]
4E8M	3.50	K^+^/Mg^2+^	Ligand-free	Yes	OiD1-5	[30]
4E8P	3.28	Rb^+^/Mg^2+^	Ligand-free	Yes	OiD1-5	[30]
4E8R	3.36	Cs^+^/Mg^2+^	Ligand-free	Partial (nonfunctional monovalent ion)	OiD1-5	[30]
4E8Q	2.84	Tl^+^/Mg^2+^	Ligand-free	Yes	OiD1-5	[30]
4E8N	2.96	NH_4_^+^/Mg^2+^	Ligand-free	Yes	OiD1-5	[30]
4E8V	3.99	K^+^/Ba^2+^	Ligand-free	No (nonfunctional divalent ion)	OiD1-5	[30]
4FAX	3.10	Na^+^/Mg^2+^	Ligand-free	No (nonfunctional monovalent ion)	OiD1-5	[30]
4FB0	3.22	K^+^/Mg^2+^	Ligand-free	Partial (active site mutant)	OiD1-5-C377G	[30]
4E8K^b^	3.03	K^+^/Ca^2+^	SER (pre)	No (nonfunctional divalent ion)	OiD1-5	[30]
4E8T	3.34	K^+^/Ca^2+^	SER (pre)	No (nonfunctional divalent ion)	OiD1-5	[30]
4FAW	2.70	K^+^/Mg^2+^	SER (post)	Yes	OiD1-5	[30]

^a^ Additional PDB entries 3BWP, 3EOH and 3G78 represent the same form of the group II intron in the state following 3′-exon hydrolysis as 3IGI, which results from the most recent refinement. These depositions were obtained by applying different integration and refinement procedures against the same set of experimental crystallographic data.

^b^ PDB entry 3EOG represents the same form of the group II intron in the state preceding the SER reaction as 4E8K, but was solved at a lower resolution (3.39 Å) using construct OiD1-6 [31].

PDB: Protein Data Bank.

The purpose of this review is to provide a framework for classifying these new structures and understanding them in the context of the splicing cycle. After providing a brief summary of all available 3-D structures of the group II introns, the catalytic cycle will be outlined in a step-by-step fashion. Each catalytic event will be presented in a way that highlights structural details while describing the experimental strategy used to capture each state crystallographically. Finally, the implications of all group II intron structures for interpreting spliceosomal function will also be discussed.

### Overview of available group II intron structures

Five different constructs have been used for crystallizing the group II introns to date. They all correspond to the *Oceanobacillus iheyensis* group II intron. Its wild type sequence was initially modified by adding a GAAA tetraloop to the terminus of the D2 stem, by inserting an RNA hairpin in place of D4, by truncating the D6 stem to about half its length, and by supplying native exons at the 5′- and 3′-ends [26]. These modifications resulted in the construct named here OiD1-6. From OiD1-6, two other constructs were derived, specifically by mutating the catalytic residue G359 to adenosine (construct OiD1-6-G359A, [31]), or by removing D6 and the flanking exons (construct OiD1-5, [30]). Finally, from OiD1-5 the construct Oi5eD1-5 was obtained by adding the short 5′-exon sequence UUAU at the 5′-end, and the construct OiD1-5-C377G was obtained by a point mutation at the catalytic position 377 [30].

Using these five constructs, 17 different structures of the *O.i.* group II intron have been published [26,27,29-31] (Figure 1, Table 1). All of these structures are highly isomorphous to each other, with pairwise root-mean-square deviation (RMSD) values in the range of 0.6 Å to 1.5 Å. Their high similarity shows that the overall intron scaffold does not undergo major structural changes during the splicing cycle. However, the active site elements do show distinctive features in each structure and five different stages of forward and reverse splicing can be discerned.

1. The precatalytic state is represented by structures 4DS6 (3.64 Å resolution [29]), and 4FAQ (3.11 Å resolution [30]) – the four-character codes are the Protein Data Bank identifiers.

2. Two structures describe conformational rearrangements that occur between the first and second splicing steps. These are 4FAR (2.86 Å resolution) and 4FAU (2.87 Å resolution) [30].

3. The postcatalytic state of the intron is represented by structure 3IGI (3.13 Å resolution) [26].

4. Seven structures reflect the ligand-free, linear form of the intron. These mimic the state of the ribozyme that is released after exon ligation, and were obtained using construct OiD1-5 crystallized in the presence of different metal ions: K^+^/Mg^2+^ (4E8M, 3.50 Å resolution), Rb^+^/Mg^2+^ (4E8P, 3.28 Å resolution), Tl^+^/Mg^2+^ (4E8Q, 2.84 Å resolution), Cs^+^/Mg^2+^ (4E8R, 3.36 Å resolution), NH_4_^+^/Mg^2+^ (4E8N, 2.96 Å resolution), Na^+^/Mg^2+^ (4FAX, 3.10 Å resolution), and K^+^/Ba^2+^ (4E8V, 3.99 Å resolution) [30]. A ligand-free form was also obtained for the functionally impaired C377G mutant (4FB0, 3.22 Å resolution). Most of the ligand-free structures represent active (K^+^/Mg^2+^, Rb^+^/Mg^2+^, Tl^+^/Mg^2+^, NH_4_^+^/Mg^2+^) or partially active (Cs^+^/Mg^2+^) states that mimic the retrotransposable form of the intron before it binds target substrates [30].

5. Four structures correspond to the retrotransposable form of the intron after target substrate binding. These structures were obtained by crystallizing the spliced (OiD1-6) or ligand-free (OiD1-5) intron with oligonucleotides that mimic ligated exons. They are 3EOG (3.39 Å resolution) [31], 4E8K (3.03 Å resolution) [30], 4E8T (3.34 Å resolution) [30], and 4FAW (2.70 Å resolution), respectively [30].

### The precatalytic state

Upon transcription, the *O.i.* group II intron folds spontaneously into a stable tertiary structure, forming a ribozyme that is highly reactive in the presence of Mg^2+^[26]. Therefore, to trap the intron in its precatalytic state crystallographically (Figure 1A1), it was necessary to deactivate the intron and prevent hydrolysis at the 5′-splice site. Two different inactivation methods have been used, namely site-directed mutagenesis [29] and metal-ion replacement [30].

The first approach (structure 4DS6) involves mutation of an invariant residue (G359) belonging to the catalytic triad motif in D5 [32-36]. Since G359 is part of a helix, in which it forms a G•U wobble pair with the partner strand, adenosine was chosen to substitute guanosine and form an A-U pair. Considering that the atoms shaping the intron active site are primarily backbone oxygen atoms, the G359A mutation was expected to cause only minimal modification of the RNA structure [29]. Indeed, in comparison to the wild-type intron, the structural perturbation in the mutant is very limited (overall RMSD = 1.2 Å). As expected, the mutation permits visualization of the 5′-splice junction. Constrained by the tight base pairing of the 5′-exon to EBS1, the junction adopts a sharp kink and forms an unusually small angle of approximately 50° between the two phosphate groups that flank the scissile phosphate [30]. Surprisingly, however, the perturbation of the active site induced by the G359A mutation was sufficient to prevent binding of catalytic metals, which explains why activity is abolished almost completely [29]. The cause of this loss of metal ion binding was explained by later studies, which elucidated the network of interactions that anchor metals in the core [30].

**Figure 3:**
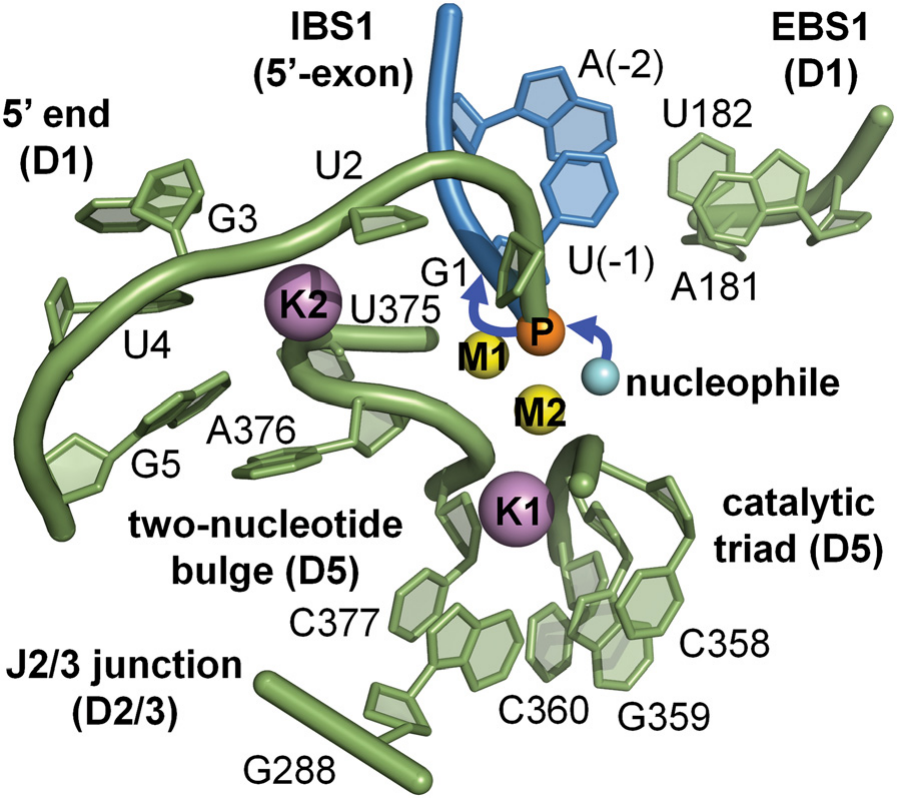
**Precatalytic state and position of the reactants for group II intron splicing.** The structure of the precatalytic state in the presence of Ca^2+^/K^+^ (4FAQ, [30]) allows the identification of all essential reactive elements. The active site scaffold (green cartoon representation) is composed of highly conserved motifs (J2/3 junction, catalytic triad, two-nucleotide bulge). The intron 5′-end connects to the 5′-exon (blue cartoon representation) via the scissile phosphate (orange sphere) and forms the 5′-splice junction. The base-paired helical structure formed by the intron and the exon binding sites (IBS1, EBS1) determines the fidelity of splicing. Finally, a four-metal heteronuclear center formed by Mg^2+^ ions (M1-M2, yellow spheres) and K^+^ ions (K1-K2, violet spheres) promotes catalysis, by correctly orienting and activating the reacting groups, including a water molecule (cyan sphere) that acts as the putative nucleophile in the reaction.

The second approach for trapping the precatalytic state (structure 4FAQ) involved the use of Ca^2+^, a structural but nonfunctional analogue of Mg^2+^. Ca^2+^ has long been known to act as an inhibitor of Mg^2+^-dependent enzymes [37] and it is also known to inhibit group II introns [38]. Ca^2+^ possesses a bigger ionic radius relative to Mg^2+^, and it does not facilitate the formation of the trigonal bipyramidal transition state at phosphorus that is typical for enzymes that catalyze phosphodiesterase S_N_2 reactions [39-42]. Although its physical-chemical properties are different from those of Mg^2+^ – Ca^2+^-bound structures must be interpreted with caution – several informative structures of endonucleases were solved in their precatalytic state by replacing Mg^2+^ with Ca^2+^[42-44]. Under these conditions, the overall intron and the geometry of its active site are not significantly affected (overall RMSD = 0.84 Å between structure 4FAR obtained in the presence of Mg^2+^ and structure 4FAQ obtained with Ca^2+^). Therefore, the Ca^2+^-bound structures opened up the way for visualizing all reactants in place for catalysis, including the metal center, the splice junction, the catalytic triple helix and the nucleophilic water molecule (Figure 3).

Taken together, the structures of the precatalytic state establish how the intron mediates two essential attributes of splicing, namely efficiency and fidelity, using the EBSs and a four-metal heteronuclear center.

**Figure 4:**
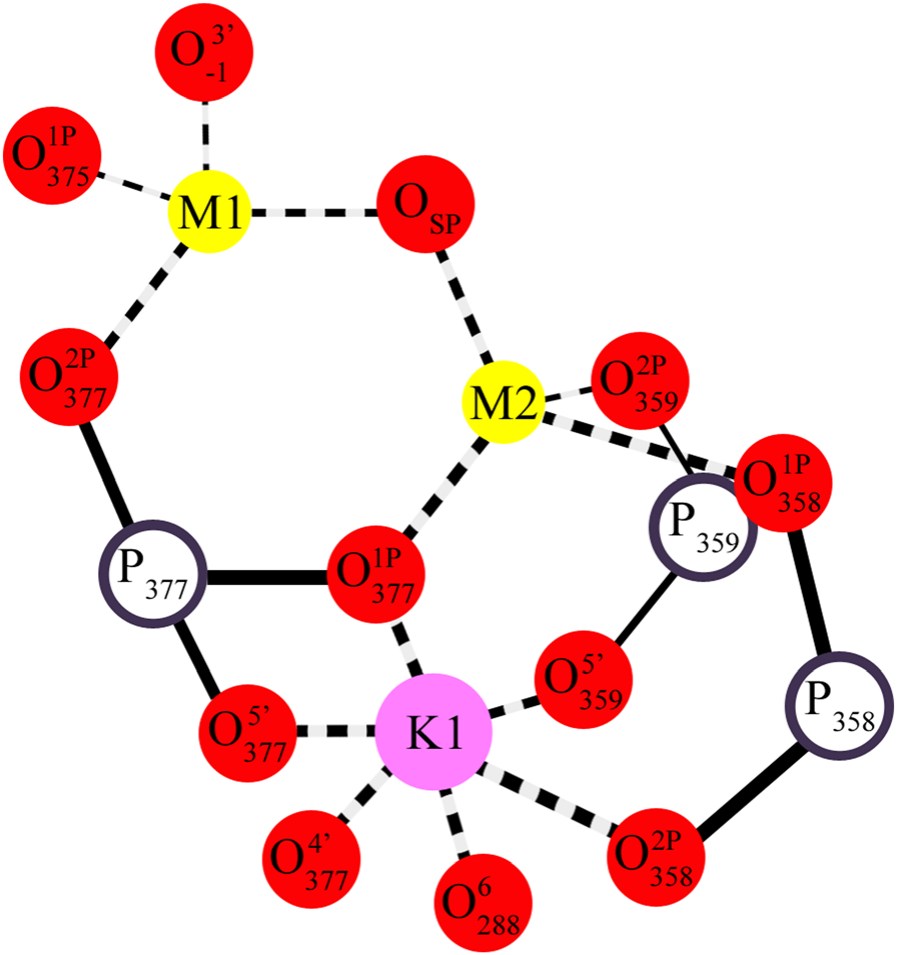
**A metal cluster in the intron active site.** Within the active site four-metal heteronuclear center (see Figure 3), three ions (M1-M2-K1) are reciprocally interconnected by single oxygen atoms. They form a *bona fide* cluster similar to organic phosphorus oxide clusters [45], with 13 vertices contributed by Mg^2+^, K^+^ and oxygen and phosphorus atoms. In the figure, magnesium (M1 and M2) is shown as yellow spheres, potassium (K1) as violet spheres, phosphorus (P) as white spheres with a purple border and oxygen (O) as red spheres. The subscript indexes are the number of the corresponding residue in the *O.i.* group II intron (the negative number is for the 5′-exon residue), while the superscript indexes represent the atom number. Continuous lines represent covalent bonds while dashed lines represent coordinative bonds. SP: scissile phosphate.

Splicing efficiency is tightly linked to the architectural organization of the metals in the active site. Four metals have been shown to be involved in catalysis [30]. Two (M1-M2) are obligate divalent ions occupied by Mg^2+^*in vivo*, while the other two (K1-K2) are monovalent ions, likely occupied by K^+^*in vivo*. Furthermore, M1-M2-K1 are interconnected by single oxygen atoms and therefore they form a *bona fide* KMgO metal cluster [30]. These ions are interconnected by three hexagonal rings of interatomic bonds, as in other organic clusters involving phosphorus (III) and phosphorus (V) oxides, but possessing 13 vertices (Figure 4, [45]). The formation of such a cluster results in a specific and highly constrained local architecture. The interconnection between the metals explains why the entire metal center is so easily disrupted when the active site residues adopt a conformation that shifts the position of the metal ion ligands, and which differs from the catalytic triple helix (*vide infra*). At the same time, the apparent rigidity of the properly assembled cluster mediates tight binding of the metals to the active site even in the absence of ligands (*vide infra*), a property that makes group II introns efficient mobile genetic elements.

By contrast, splicing fidelity is linked to the appropriate pairing of EBS-intron binding site (IBS) elements. Structure 4DS6 shows that the formation of the EBS1-IBS1 interaction is sufficient to place the 5′-splice junction correctly in the active site even if other elements, including the metal cluster, are not well positioned. Also the intron structures solved using OiD1-5 in a ligand-free state (*vide infra*) provide an illustrative example of how splicing fidelity is achieved. Specifically, OiD1-5 possesses a short poly-G sequence (GGG) at its 5′-end, and this fails to interact with the EBS1 site. This sequence is artificially inserted immediately downstream of the T7 promoter in order to enhance the yield of *in vitro* transcription by T7 RNA polymerase [46-48]. Because the GGG sequence is different from that of the native 5′-exon (UUAU) and therefore does not possess any complementarity to EBS1 (AUAA, Figure 2), the 5′-splice junction in those structures is flexible and completely excluded from the active site, even when the catalytic metal center is intact [30]. Thus, EBS1 is highly specific in choosing its partner nucleotides at the 5′-splice site, as supported also by biochemical evidence [49].

### Putative position of the branching nucleotide

**Figure 5:**
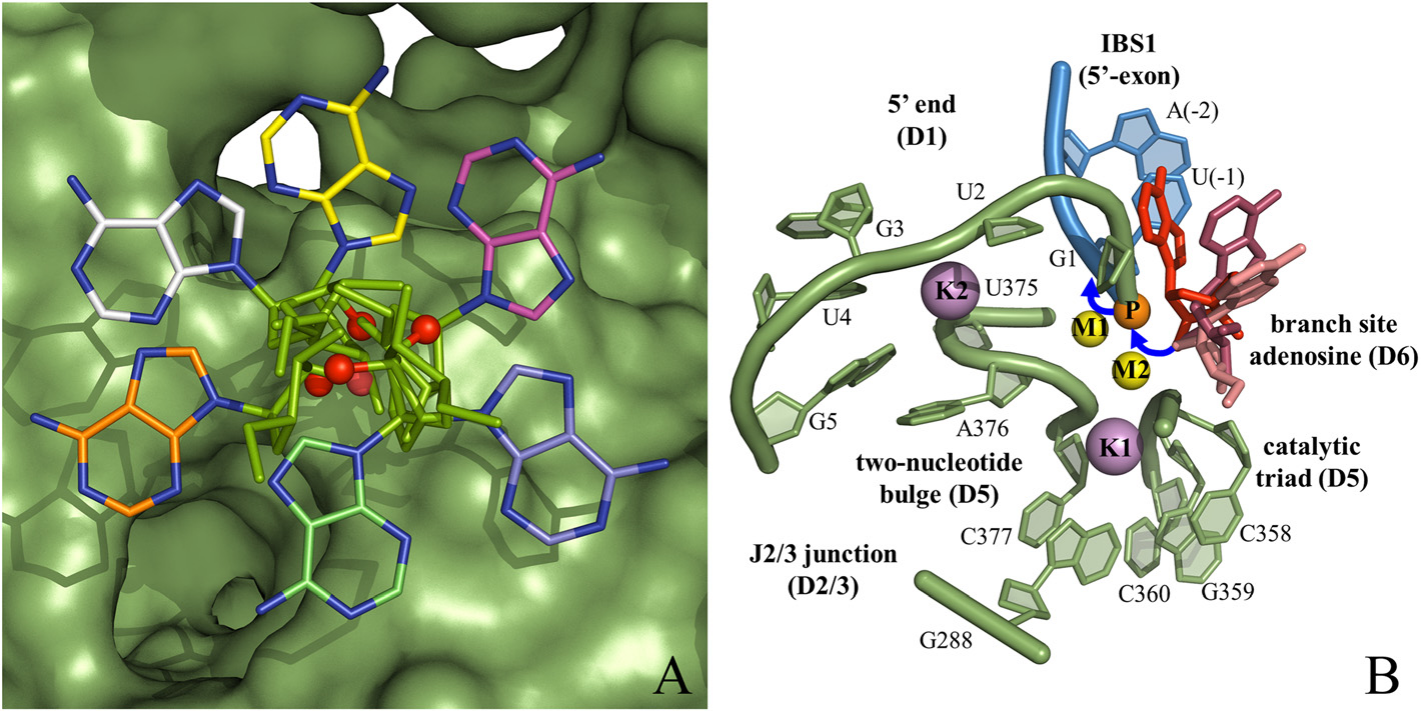
**Putative position of branching nucleotide.** Possible locations of branching adenosine were modeled by manually docking the residue into the intron active site. Panel **A** shows a series of sterically allowed conformations (adenosine is represented by sticks, carbon atoms of the base are in various colors, the ribose moiety and the surface of the intron active site are in green). Among these conformations, only a subset of structures orients the nucleophilic 2′-OH group (red spheres in panel **A**) in line with the scissile P-O bond, in the same position as the water molecule that acts as the nucleophile for hydrolytic splicing (see Figure 3). The latter subset of structures is shown in panel **B** (oriented and color coded as in Figure 3).

No crystallographic data is available to describe the position of the 2′-OH group of the branching residue involved in splicing by transesterification (Figure 1A2). However, its position can be inferred based on the identification of the nucleophile in the structure that describes the hydrolytic reaction (4FAQ) [50]. Certainly, predicting the correct position of this branching residue in the absence of experimental data is difficult because the nucleophilic adenosine and D6 form few interactions with the rest of the intron [51]. It is known that the branching nucleotide needs to be an adenosine to achieve maximal splicing efficiency, but this residue does not control the fidelity of the reaction and other nucleotides are also compatible with branching albeit with low efficiency [51]. Indeed, in the spliceosome, the splicing machinery corresponding to group II introns in eukaryotes, the branch site has been studied extensively and it has been shown that the precise location of the branch site is not always tightly fixed [52,53]. Additionally, the branch site nucleophile is usually bulged or dynamic within the D6 stem but even this is not an absolutely conserved requirement [51,54,55]. However, despite these uncertainties, it is possible to model the position of D6 using the steric constraints imposed by the other active site elements and by the geometrical requirements of the S_N_2 reaction that is typical of group II intron splicing (Figure 5). These models show that a limited number of conformers are sterically allowed in which the trigonal bipyramidal geometry is maintained.

### A conformational transition to the second splicing step

After the first splicing step, the intron active site becomes rearranged before performing the second transesterification reaction. Specifically, D5 is known to become rearranged, thanks to the flexibility of its two-nucleotide bulge motif [12,56], while D6 toggles between an active state coordinated to the κ-coordination loop or the D1C helix, and a silent state forming the η-η' interaction with D2 [21,28,57]. However, biochemical experiments such as crosslinking studies [24] and all available crystal structures suggest that a group II intron possesses only one catalytic site for both the first and second splicing steps [12,24,58].

Given these observations, one might assume that the reactants for the second splicing step, which remains crystallographically uncharacterized, are already aligned correctly for catalysis in the precatalytic state. However, this is not the case, as long-range interactions involving the second splicing step reactants have been shown to form only between the first and the second splicing steps, or to affect selectively the second and not the first splicing step (that is, the γ-γ' interaction, the interaction between the first and the penultimate intron nucleotides, the IBS3-EBS3 interaction and the η-η' interaction [59-61]). Furthermore, in the structures, the nucleophile of the first splicing step is located near the EBS3 site, in the identical position that must be occupied by the 3′-splice junction during the second splicing step [30]. Therefore, there is also a structural incompatibility that prohibits accommodation of all reactants in the same active site at once. Consequently, a rearrangement of the active site between the splicing steps is likely to happen.

In light of recent structures, more detailed hypotheses about such a rearrangement can be proposed. The structures suggest two types of conformational rearrangements, one involving a movement of the hydrolyzed scissile phosphate (Figure 1A1), the other a movement of the J2/3 junction and the two-nucleotide bulge (Figure 1B). The first conformational rearrangement, which directly follows 5′-exon cleavage, was visualized by crystallizing Oi5eD1-5 in the presence of the physiological, catalytically functional ions Mg^2+^ and K^+^ (structure 4FAR, reference [30] and Figures 1 and S1 therein). Upon hydrolysis, which occurs during the crystallization process, the 5′-exon maintains coordination to M1 through its 3′-OH group and is not displaced significantly from its binding site, as expected since the 5′-exon is the nucleophile of the second splicing step. Instead, hydrolysis induces relaxation of the kinked RNA backbone at the 5′-splice junction and the hydrolyzed scissile phosphate is released from the active site. Specifically, the free phosphate is displaced by about 4 Å, where it interacts directly with the K2 site, which evidently plays a direct role in organizing, and potentially liberating, splicing products. The second conformational rearrangement was visualized in a structure of Oi5eD1-5 solved in the presence of Li^+^/Mg^2+^ (4FAU) [30]. In this structure, the 5′-exon has undergone hydrolysis and one observes an equilibrium between two conformations in the active site: the catalytic triple helix conformation and an inactive toggled conformation. The conformational change involves two residues in the J2/3 junction (G288-C289) and one residue in the two-nucleotide bulge (C377, D5), all known to be dynamic elements of group II introns [12,58]. In the inactive toggled conformation, which is visualized most clearly when the intron is crystallized in a buffer of Na^+^/Mg^2+^ (structure 4FAX, see reference [30] and Figure 4 therein), G288 rotates by about 90° around an axis connecting its C5′ and C3′ backbone atoms, while the cytosine moiety of C377 rotates by about 70° around the glycosidic bond. Both residues in the inactive toggled conformation are stabilized by a new network of interactions. Among these, two involve the 2′-OH groups of both residues, which do not form any interactions in the triple helix conformation typical of the precatalytic state. These interactions are particularly interesting because the two hydroxyl groups had previously been demonstrated to be important in catalysis using biochemical methods, but their role was unclear until now [32,34]. In addition to disrupting the triple helix, the conformational rearrangement also moves the RNA ligands that are essential for anchoring the M1-M2-K1-K2 metal center. This causes the interactions between catalytic ions and the 5′-splice junction to be broken and facilitates the release of the latter.

In summary, therefore, a concerted conformational rearrangement appears likely to promote the transition to the second splicing step. Considering the central role of the residues involved in the rearrangement, we cannot exclude the notion that the inactive toggled intron conformation might also occur at other points of the splicing cycle, and we would like to suggest two scenarios to support this hypothesis. First, the inactive toggled conformation may represent an intermediate conformation that occurs while the intron folds into its active, precatalytic state. This hypothesis is supported by the fact that a mutant designed to stabilize the inactive toggled conformation (C377G) shows a tenfold reduction in the rate of the first splicing step in addition to its pronounced defect in the second splicing step (see reference [30] and Figure S5 therein). Second, the opening of the triple helix and the consequent disruption of the active site metal cluster may be important for successfully terminating the splicing cycle, when the ligated exons must be released from the active site to form a free intron. The inactive toggled conformation would prevent ligated exons from being rehydrolyzed through SER, which is a prevalent *in vitro* side-reaction that represents a major problem for productive splicing *in vivo*.

### Second splicing step

The second splicing step remains an important area for future structural studies, as it has not been fully elucidated by existing structures. Two sets of structures would be required to describe its mechanism at a molecular level, namely the structure of the state preceding cleavage of the 3′-splice junction and the structure of the postcatalytic state. While the latter can be represented by structure 3IGI (Figure 1C), which corresponds to the postcatalytic linear intron harboring products of the splicing reaction in its active site [26,27]; the former structure is not yet available and can only be deduced from modeling exercises (Figure 1C).

**Figure 6:**
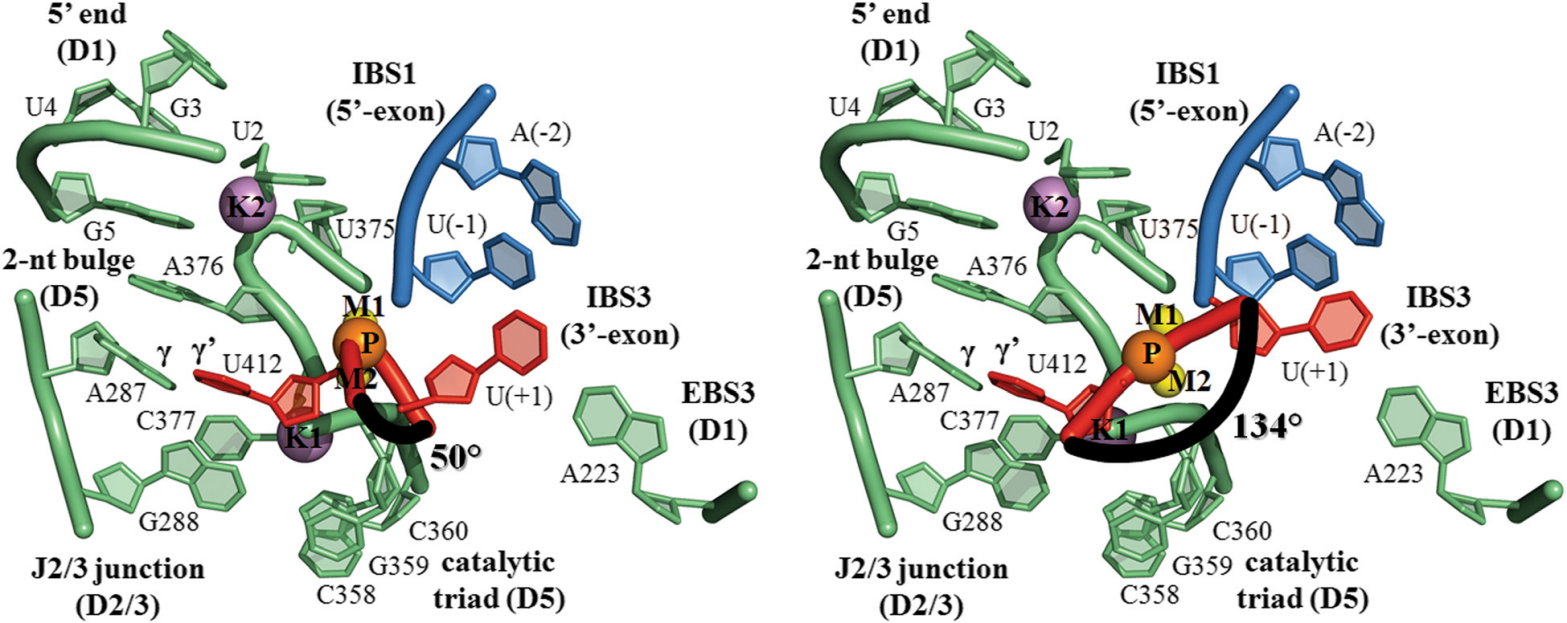
**Models of the 3′-spliced junction.** The junction (red ribbon) is constrained into the active site by two sets of interactions: the γ-γ' interaction that joins the last intron nucleotide (U412) with A287 in the J2/3 junction (intron nucleotides are depicted in green), and the EBS3-IBS3 interaction that joins the first nucleotide of the 3′-exon (U+1); with A223 in D1. The scissile phosphate (P) presents its pro-S oxygen to the catalytic metals (M1-M2, yellow spheres). The backbone of the junction may be kinked (angle of 50° between the two phosphate groups that flank the scissile phosphate, left panel, [29]) or elongated (angle of 134°, right panel).

Specifically, modeling the geometry of the 3′-splice junction before cleavage can be done on the basis of the following considerations. First, the position of the 3′-OH group of the 5′-exon, which acts as the nucleophile on the 3′-splice junction, can be derived from structures 4FAR and 4FAU (see above and [30]). These structures show that, after the first splicing step, the 5′-exon does not change its position within the active site and that it remains bound to the EBS1 site. Second, the position of the catalytic metal center can be deduced from the structures of the postcatalytic states of the intron (3IGI, 3EOG, 4E8K, 4E8T and 4FAW [26,30,31] and *vide infra*). These structures show that, after catalysis, the metals occupy identical positions as in the precatalytic state (see above). Therefore, it can be expected that in the second splicing step the metal center reassembles in the same conformation as in the first splicing step, after being transiently disrupted by the swinging and toggling mechanism described above [30]. Third, the structure of three residues around the 3′-splice junction (penultimate and last intron nucleotides and first exon nucleotide) can be modeled *de novo*, based on the known positions of other intron residues with which they engage specific tertiary interactions previously identified by biochemical experiments [60-62]. The penultimate intron nucleotide engages in an interaction with G1 [62], whose position can be derived from structure 4FAR. The last intron nucleotide forms the γ-γ' interaction [61] with A287 (J2/3 junction), whose position is determined by structures 4DS6, 4FAQ, 4FAR, 4FAU, 4E8M, 4E8P, 4E8R, 4E8Q, 4E8N, 4E8V, 4E8V, 4FAX, 4FB0, 4E8K, 4E8T and 4FAW. Finally, the first exon nucleotide (IBS3 site) base-pairs with residue A223 (EBS3) [60], and the structure of this IBS3-EBS3 interaction can be derived from structures 4E8K and 4E8T. Finally, the model of the 3′-splice junction must also consider that the scissile phosphate prefers to adopt an Rp stereochemical configuration before nucleophilic attack, as determined by phosphorothioate substitutions [63]. Based on these structural and biochemical constraints, we modeled the reactants of the second splicing step. Here we present two possible models, both compatible with available biochemical data and possessing favorable structural geometry. In the first case, which has already been proposed [29], the 3′-splice junction is modeled in a kinked conformation. In the other case, the junction adopts an extended conformation instead (Figure 6).

### Postcatalytic ligand-free state

Upon completion of the splicing reaction, the ligated exons are released from the active site and the free intron is liberated in a linear or lariat form. While the structure of the lariat form is not yet available, many structures have been obtained for the linear form (4E8M, 4E8P, 4E8R, 4E8Q, 4E8N, 4E8V, 4FAX and 4FB0; see Figure 1D) [30].

To obtain the structures of the excised intron in a ligand-free state (that is, without any bound exons or ligated exons), it was necessary to prevent co-crystallization of exon-like fragments deriving from the splicing reaction and from self-degradation of the intron [26]. To this end, we utilized construct OiD1-5, which folds spontaneously during transcription *in vitro*, and adopts a homogeneous, active conformation after purification, yielding a free, multiple-turnover ribozyme that is a good mimic for the postcatalytic state of the intron [30]. The ligand-free intron structures are nearly identical to the available ligand-bound ones, which is a fairly typical case for protein enzymes and ribozymes that catalyze two-metal ion phosphodiester cleavage reactions [30]. All residues are visible in the electron density and only the EBS1 site is slightly disordered, as expected given the absence of base-pairing with a corresponding IBS1 sequence. Despite their overall similarity to the ligand-bound states of the intron, the ligand-free intron structures show remarkable features, particularly in terms of the catalytic metal ions.

First, the ligand-free structures show that, even in the absence of K^+^, monovalent ions like Tl^+^, Rb^+^, Cs^+^, Na^+^ and NH_4_^+^, and divalent ions like Ba^2+^ can support the correct folding of the intron scaffold. Therefore, these structures unambiguously reveal the identity of numerous important metal binding sites. These observations demonstrate a remarkable adaptability of group II introns, and potentially other large RNA molecules, to different metal ions. This is important given that metal ions are very useful tools for studying large RNAs, not only crystallographically [30,64], but also spectroscopically [65,66] and biochemically [67].

Second, the ligand-free structures show that the catalytic metal center M1-M2-K1-K2 is correctly bound within the active site when the intron is crystallized in the presence of physiological ions (Mg^2+^/K^+^), or any other ions that support chemical catalysis. This observation is surprising considering that the metals – in particular M1 and M2 – are less tightly coordinated and more exposed to the solvent in the absence of the exons. Indeed, in the ligand-free structures M1-M2 are bridged by a water molecule that occupies the position of the scissile phosphate oxygen [30]. This water molecule is therefore likely to represent an important element in the ligand-free active site, because it completes the KMgO cluster. The integrity of the active site in the ligand-free intron supports the observation that this ribozyme is a highly efficient retrotransposable element.

### SER and retrotransposition

The structure of the empty, ligand-free intron sets the stage for understanding the mechanism of its retrotransposition into genomic DNA or into RNA (Figure 1E) [68]. The first retrotransposition step (which is a reverse-splicing reaction) is thought to be approximated *in vitro* by the spliced-exon reopening reaction, where ligated exons are bound and then attacked by the free intron, because the chemistry of the two reactions is known to be identical [13-15]. Both the precatalytic and the postcatalytic states of the SER reaction have now been characterized crystallographically using RNA substrates (structures 3EOG, 4E8K, 4E8T and 4FAW [30,31]).

The precatalytic state of SER was first visualized in 2008, when a self-spliced intron was co-crystallized with an oligonucleotide mimicking ligated exons (structure 3EOG) [31]. In another approach to visualizing the precatalytic state of SER, construct OiD1-5 was co-crystallized in the presence of Ca^2+^ with an oligonucleotide that corresponds to the sequence of native ligated exons (structures 4E8K and 4E8T) [30]. These latter structures revealed the presence of an intact active site, whose geometry is highly reminiscent of that of the precatalytic state preceding 5′-exon hydrolysis. The scissile phosphate of the substrate is located between the M1 and M2 sites, presenting the pro-S oxygen atom at approximately 2 Å from each of the two metals. The stereochemistry of the scissile phosphate in the structure is thus in perfect agreement with previous biochemical experiments that had predicted a preference for the pro-S configuration on the basis of phosphorothioate substitutions [69]. Moreover, the 5′-exon portion of the oligonucleotide tightly binds to the EBS1 site, while the 3′-exon nucleotide shows a well-defined Watson–Crick base-pairing only for the uridine at the scissile position (IBS3) with the corresponding EBS3 adenosine. M1 coordinates to the leaving group (the 3′-OH of the nucleotide in 5′ to the scissile phosphate), while M2 coordinates to the scissile phosphate oxygen, in agreement with the two-metal ion mechanistic hypothesis [70]. By contrast, the structure of the post-hydrolytic state of SER was obtained using the OiD1-5 construct, bound to the same oligonucleotide used for solving 4E8K and 4E8T, but co-crystallized in the presence of physiological ions Mg^2+^ and K^+^ (structure 4FAW) [30]. This structure currently represents the structure of the intron at the highest resolution ever achieved (2.7 Å) and so far the highest resolution structure of a noncoding RNA longer than 200 nucleotides, except for the ribosomal subunits. In this structure, the 5′-exon portion of the oligonucleotide is visible in the electron density, as it forms base pairs with the EBS1 binding site in the same position as in the pre-hydrolytic state. By contrast, the 3′-end has been released and, as happens for ligand-free structures, the KMgO cluster is completed by a water molecule bound between M1 and M2.

The structures of the IBS-EBS interactions and of the metal center of the SER reaction are particularly significant, because they help in understanding the mechanism of the second splicing step, as discussed above. Furthermore, a solvent molecule coordinated by C358 in the catalytic triad and by M2 can also be identified in the precatalytic state (structures 4E8K and 4E8T) at about 3.2 Å from the scissile phosphate, in a direct line with the scissile P-O bond [30]. This positioning, which is identical to that of the nucleophile for the first splicing step, suggests that this solvent molecule likely represents the reaction nucleophile of the SER reaction. Therefore, it represents the most likely location occupied by the nucleophile of the first reverse-splicing step, namely the 3′-OH group of the last intron nucleotide. These observations further corroborate the hypothesis of a single major active site for group II introns [24] and shed light on the molecular mechanism of the retrotransposition event. Certainly, to obtain a more complete visualization of the reverse-splicing reaction, it will be necessary to crystallize the intron in complex with DNA substrates.

### Implications for the spliceosome

Besides revealing the molecular mechanism of different stages of the intron splicing cycle, the structures described so far also provide new evidence to support the idea that group II introns may be functionally and structurally related to the spliceosome [6-8]. Therefore, we will briefly discuss how the intron structures contribute to a deeper understanding of spliceosomal architecture and function.

Group II introns and the spliceosome have many strong analogies. Sequence conservation analyses revealed precise correspondence of active site motifs in the two systems [71]. Specifically, the catalytic triad is well conserved within intron D5 and in the spliceosomal snRNA subunit U6 [72], the J2/3 junction (intron D2-3) corresponds to residues in the conserved spliceosomal ACAGAGA box (U6) [71], and the two-nucleotide bulge motif (intron D5) is likely to correspond to bulged residues either in the internal stem loop of U6 (U80, [71,73]) or in U2-U6 helix I (A25, [30,74]). Mutations at any of these conserved positions have similar effects in the two systems [14,58,75,76]. Besides sequence similarities, the two macromolecules also share similar preferences for the stereochemical configuration of the scissile phosphate in the two splicing steps [15,63,77]. Moreover, the metal ion requirements are strikingly similar in both the intron and the spliceosome. Not only are both machineries selectively dependent on magnesium as a divalent ion [4,78], but they are also both tightly controlled by monovalent ions, that is, potassium [50,79]. Finally, both macromolecules are known to pause in transiently inactive states to regulate the transitions between the different splicing steps [30,80].

In the light of these analogies, it seems plausible to believe that the mechanistic details learned from the new intron structures may be pertinent for spliceosomal splicing. In particular, the structural arrangement of the active site motifs and the reactants, the identity and the coordination of the metal ions in the catalytic heteronuclear center, and possibly the dynamics of the conformational toggling observed for the group II intron may have similar correspondence also in the spliceosome. Two specific hypotheses have been proposed, each agreeing with different sets of experimental data and differing in the choice of the toggling residues and in how the spliceosomal elements are positioned in the active site [30]. Other scenarios are also possible and further studies on the spliceosome are required to obtain a more detailed representation of its active site.

Certainly, at present, it is very hard to picture with atomic precision a similarity between the approximately 150-kDa monomeric group II intron ribozyme and the approximately 12-MDa, heteromultimeric spliceosomal ribonucleoprotein. Recently, though, a significant milestone in this direction has been achieved with the determination of the crystal structure of Prp8, a spliceosomal component that interacts directly with all active site elements [81]. Importantly, the Prp8 structure suggests that none of the protein motifs possess catalytic activity, thus reinforcing the current belief that spliceosomal chemistry is performed by the RNA subunits [81]. Even more interestingly, the structure reveals that Prp8 folds around an overall positively charged cavity whose dimensions exactly correspond to the conserved RNA components within the group II intron active site [81]. Evolution seems to have substituted the group II intron scaffold, which is provided by the noncatalytic intron domains (predominantly D1), with the protein scaffold of Prp8, presumably to achieve a finer regulation of splicing fidelity and a more elaborate coordination of the interaction network with other spliceosomal components and regulatory factors. Within this shell, catalytic elements similar to those of a group II intron (for example, U6) are still believed to reside in the core of the spliceosome, suggesting that an RNA element similar to the group II intron D5 is conserved from bacteria to humans.

Overall, the combination of all new structures of group II introns and spliceosomal components reinforces the hypothesis that the two systems may share a common catalytic core and a common mechanism for arranging their reactants and controlling the transitions between the chemical splicing steps.

## Conclusions

The large collection of available group II intron structures has recently brought our understanding of splicing mechanism to a new level.

Future work is now likely to focus on the characterization of D6, and the structure of conformational states that participate in branching. Hopefully, these types of structures will reveal the position of the branching nucleotide involved in the mechanism of the first splicing step and will pave the way for visualizing the structures of the branched intron/3′-exon intermediate and of the ligand-free lariat intron. Furthermore, structures containing D6 will reveal the conformation of the 3′-splice junction in the precatalytic state, and in the state that immediately precedes the second splicing step.

Eventually, all these structural snapshots will enable the creation of a movie that depicts each stage of the splicing cycle at high resolution. These pieces of structural information will be valuable not only for understanding the reaction mechanism of group II introns, but for understanding pre-mRNA splicing in general, as group II introns share many structural and mechanistic features with their spliceosomal cousins.

## Abbreviations

EBS: Exon binding site; IBS: Intron binding site; O.i.: *Oceanobacillus iheyensis*; PDB: Protein Data Bank; RMSD: Root-mean-square deviation; SER: Spliced-exon reopening; SP: Scissile phosphate.

## Competing interests

The authors declare that they have no competing interests.

## Authors’ contributions

MM analyzed the structures, designed the model of the 3′-splice junction and wrote the manuscript incorporating contributions from all authors. SS designed the models of the branch site and the 3′-splice junction. AMP conceived and coordinated the study. All authors read and approved the final manuscript.

## Authors’ information

MM and SS are currently postdoctoral associates at Yale University. AMP is the William Edward Gilbert Professor of Molecular, Cellular and Developmental Biology and Professor of Chemistry at Yale, and a Howard Hughes Medical Institute Investigator.
